# Supplementary material for: Effectiveness and Safety of Ayurvedic Medicines in Type 2 Diabetes Mellitus Management: A Systematic Review and Meta-Analysis
Source: Front Pharmacol. 2022 Jun 8;13:821810. doi: 10.3389/fphar.2022.821810 (PMC9213670; doi:10.3389/fphar.2022.821810)
Supplement: Supplementary file 1 [file DataSheet1.zip › Supplementary Material/Forest and Funnel Plots/Trigonella foenum-graecum/Subgroup analyses- comparator/Trigonella foenum-graecum_FBG_comparator subgroup analysis.pdf]

Trigonella foenum-graecum - FBG - comparator subgroup analysis

Study	Intervention N	Intervention Mean	Intervention SD	Comparator N	Comparator Mean	Comparator SD	Mean Diff. with 95% CI	Weight (%)
**No medicine / no additional medicine**								
Kaur 2016	30	141.23	10.11	30	144.43	9.86	-3.20 [-8.25, 1.85]	9.67
Singh 2016	20	121.55	12.63	20	113.70	12.99	7.85 [-0.09, 15.79]	9.18
Ranade 2017	30	151.08	28.88	30	158.57	27.46	-7.49 [-21.75, 6.77]	7.72
Hota 2019	102	139.39	40.73	102	144.23	46.40	-4.84 [-16.82, 7.14]	8.28
Hadi 2020	24	132.60	21.21	24	142.61	29.27	-10.01 [-24.47, 4.45]	7.67
Heterogeneity: $\tau^2 = 25.54$, $I^2 = 52.71\%$, $H^2 = 2.11$							-2.18 [-8.51, 4.14]	
Test of $\theta_i = \theta_j$: Q(4) = 8.06, p = 0.09								
**Placebo**								
Gupta 2001	12	119.90	25.00	13	113.00	36.00	6.90 [-17.60, 31.40]	5.32
Lu 2008	46	122.33	25.22	23	150.97	24.50	-28.64 [-41.15, -16.13]	8.16
Rafraf 2014	44	136.00	46.44	44	158.00	64.90	-22.00 [-45.58, 1.58]	5.51
Verma 2016	77	135.70	44.87	77	151.12	48.11	-15.42 [-30.11, -0.73]	7.61
Gholaman 2018	10	185.40	17.25	10	216.10	26.32	-30.70 [-50.20, -11.20]	6.43
Kandhare 2018	59	162.80	50.72	53	170.05	57.45	-7.25 [-27.28, 12.78]	6.30
Hassani 2019	62	114.56	25.93	63	157.16	41.19	-42.60 [-54.69, -30.51]	8.26
Rashid 2019	32	113.50	7.93	32	134.58	5.40	-21.08 [-24.40, -17.76]	9.88
Heterogeneity: $\tau^2 = 119.98$, $I^2 = 75.11\%$, $H^2 = 4.02$							-21.91 [-31.39, -12.44]	
Test of $\theta_i = \theta_j$: Q(7) = 21.49, p = 0.00								
**Overall**							-13.72 [-21.97, -5.47]	
Heterogeneity: $\tau^2 = 176.36$, $I^2 = 88.37\%$, $H^2 = 8.60$								
Test of $\theta_i = \theta_j$: Q(12) = 100.62, p = 0.00								
Test of group differences: $Q_b(1) = 11.52$, p = 0.00								

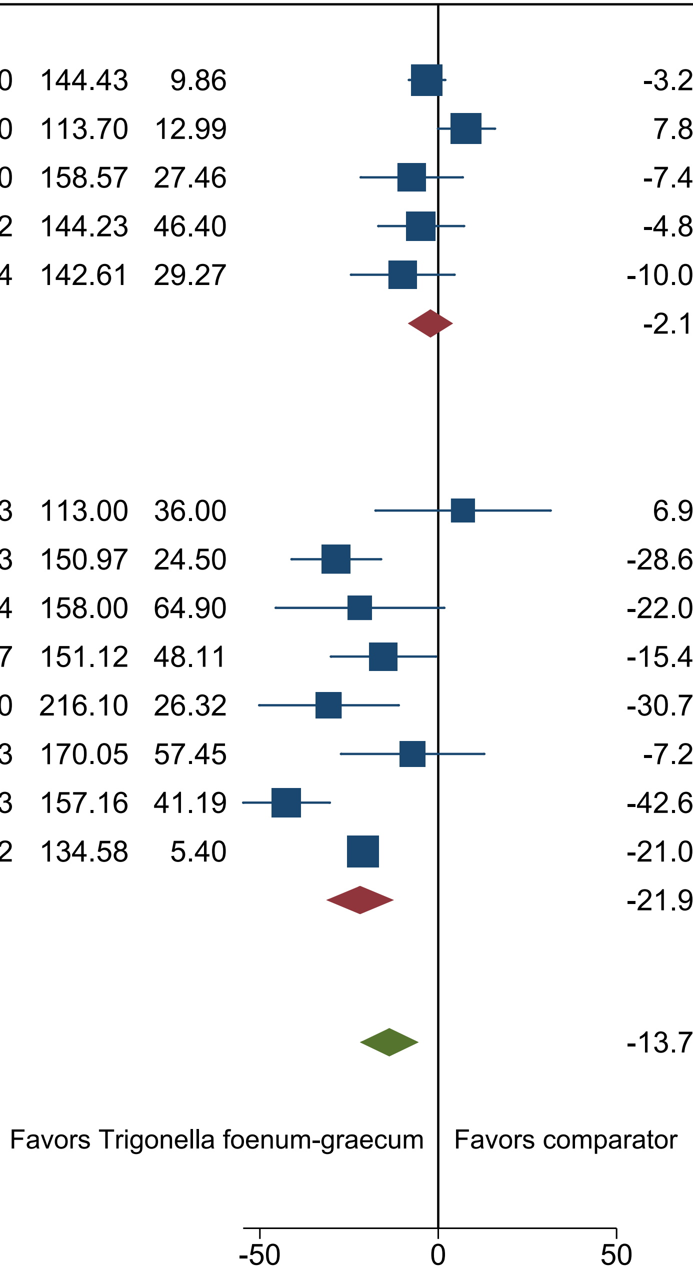

Random-effects REML model
